# Fertilization rate of crossbreeding cattle using sexing and conventional semen in different seasons in South Papua

**DOI:** 10.5455/javar.2024.k845

**Published:** 2024-12-27

**Authors:** Nurcholis Nurcholis, Lilik Sumaryanti, Apri Irianto, Syetiel Maya Salamony

**Affiliations:** 1 Department of Animal Husbandry, Universitas Musamus, Merauke, Indonesia; 2 Department of Informatic Engineering, Universitas Musamus, Merauke, Indonesia

**Keywords:** Conception rate, semen, sexing, female cattle, THI

## Abstract

**Objective:**

Fertilization rate of artificially inseminated cows using sexed and conventional semen in different seasons in South Papua.

**Materials and Methods:**

Eighty crossbred cows aged 4–4.5 years with body condition score 3.8 were divided into groups A (summer = 40 cows) and B (rainy season = 40 cows). Each cow in each season was artificial insemination (AI) using sexed frozen semen and conventional semen. Frozen semen was evaluated for post-thawing motility (PTM), cell membrane integrity, and acrosome damage before synchronization using 5 ml PGF2α plus vitamin E. Using a visual gun, we identified cows in estrus on days 4–7 post-synchronization. Pregnancy of cows was detected using N5Vet ultrasound on days 35 and 55. The interaction between season, semen type, and fertilization level was analyzed using standard error and two-way ANOVA, assisted by SPSS 21 software.

**Results:**

The wet season Temperature-Humidity Index (THI) level averaged 77.12 ± 1.19, and the summer season THI level averaged 82.67 ± 1.25. PTM quality averaged 60%–65%, viability 61%–71%, sperm membrane integrity 62%–65%, and acrosome integrity 88%–91%. Conception rates (CR) value of rainy season (*p < *0.05) with summer season. In addition, the services per conception (S/C) value in the rainy season (*p* > 0.05) is the same as in the summer. This study’s S/C and CR values were within normal limits, and the pregnancy rate reached 65%–86%. Pregnancy detection can be observed on day 35, and the fetal heartbeat is visible.

**Conclusion:**

Post-AI fertilization using conventional semen was better in all seasons. The double dose of sexed semen can increase the fertilization rate in summer.

## Introduction

Small-scale cattle farming in Merauke, South Papua, generally involves crossbreeding with the help of reproductive technology such as artificial insemination (AI) using conventional frozen semen [1]. Many cattle farmers want male calves, and this is because the selling price of AI-produced bulls is more expensive than female cows. The selection of bull offspring can be done with the application of semen sexing. Until now, the use of semen sexing in the Merauke region of South Papua as the center of beef cattle farming in the community has never been done, so the success rate is unknown. The success of AI in each region is different, mainly due to several factors, especially internal factors such as cow breeds and external factors such as frozen semen, temperature, and humidity.

In 2022, the temperature in Merauke in February-May was 26°C–28°C, while September-December was 30°C–34°C. The Temperature-Humidity Index (THI) value can be used as an indicator of stress levels in livestock [2]. A high THI value will affect the physiological response of livestock, which will impact their productivity. It is suspected that stress has an impact on fertilization rates in cattle. In addition to stress, fertilization success is influenced by several factors, including semen quality, estrus timing, AI officer skills, and temperature. Estrus detection and AI officer skills are essential factors in AI success in Merauke [3].

However, until now, the success rate of AI using sexed semen in different seasons has not been known in South Papua. Therefore, this study was to determine the fertilization rate of crossbreeding cattle AI using sexed and conventional semen in different seasons.

## Materials and Methods

### Ethical approval

This study has received ethical approval and animal welfare number 349.1/UN52.8/KL/2023 from Musamus University.

### Location and female cattle

This study was conducted in the September–December 2023 summer season (A) and January–March 2024 rainy season (B) at Semangga District, Merauke Regency, South Papua. Location selection was based on the highest number of cattle in the Merauke district (BPS, 2022). The number of AI cows was 80, divided into two seasons, each using 40 cows. The cows were 4–4.5 years old and had an average body condition score of 3.8 on a scale of 1 for very thin and 5 for very fat [4]. The main diet was fresh forage plus concentrate at a ratio of 70:30, and drinking water was provided *ad libitum*. All cows had no reproductive problems, and the veterinary officer checked the regular lambing interval.

### Collection of temperature and humidity data

Temperature and humidity data were collected consistently at 06:30 am and 17:30. Thermopro TP359 smart temperature and humidity measuring device connected to Bluetooth 80 m. The equipment was placed in the cowshed at a height of 1.5 m from the ground. Temperature and humidity measurements were conducted in two seasons: summer (September–December) and rainy (January–March), as the THI data processing refers to [5].

### Evaluation of post-thawing semen and sperm acrosome damage

The general sperm evaluation process refers to Nurcholis et al. [6]. Post-thawing evaluation of frozen semen tests sperm motility, sperm viability, sperm cell membrane, and acrosome damage. Sperm motility was evaluated by adding physiological NaCl and observed under 40× magnification. Evaluation of sperm viability with eosin-nigrosin staining in the ratio of 1 drop of sperm to 2 drops of eosin-nigrosin, observed under 100× magnification. Use of HOS-Test solution to determine the reaction of sperm as evidence of active cell membranes. Testing for sperm acrosome damage using Giemsa combined with methanol fixation [7]. All observations were evaluated under a binocular microscope (Olympus CX43) connected to a Japan monitor.

### Synchronization, AI, and pregnancy detection procedures

The cows used have been examined, are free from reproductive diseases, and have a continuous birth rate. The cows used must have given birth at least twice. Corpus luteum detection was done by rectal palpation before AI. Synchronization is done by injecting 5 ml of the hormone PGF2α plus vitamin E. Evaluation of estrus is observed 4–7 days after synchronization or based on the discharge of thick and clear mucus in the cow’s vagina. Female cows in estrus condition were AI using the ALGGO China brand AI visual gun, carried out in the morning or evening with a semen dose of 0.25 ml. In each season, the AI process used 20 sexed semen and 20 conventional semen. After the AI process, the cows were rested in the cage for 1 day. Pregnancy detection was conducted using USG N5Vet on days 35 and 55. The initial determination of pregnancy detection using ultrasound is based on [8]. The success rate of AI can be seen from the services per conception (S/C) and conception rates (CR) values as follows:


$$\mathrm{CR}\;=\;\frac{\mathrm{Number}\;\mathrm{of}\;\mathrm{pregnant}\;\mathrm{cows}\;\mathrm{with}\;1\mathrm{st}\;\mathrm{inse}\min\mathrm{ation}}{\mathrm{Number}\;\mathrm{of}\;\mathrm{acceptor}}\;\times \;100\%$$

$$S/C\;=\;\frac{\mathrm{Number}\;\mathrm{of}\;\mathrm{AI}}{\mathrm{Number}\;\mathrm{of}\;\mathrm{pregnant}\;\mathrm{cows}}$$

### Data analysis

The interaction between season, semen type, and fertilization rate was analyzed by means of squared and standard error using two-way ANOVA using SPSS 21 software.

## Results

### Daily temperature, humidity, and THI

**Figure 1.**
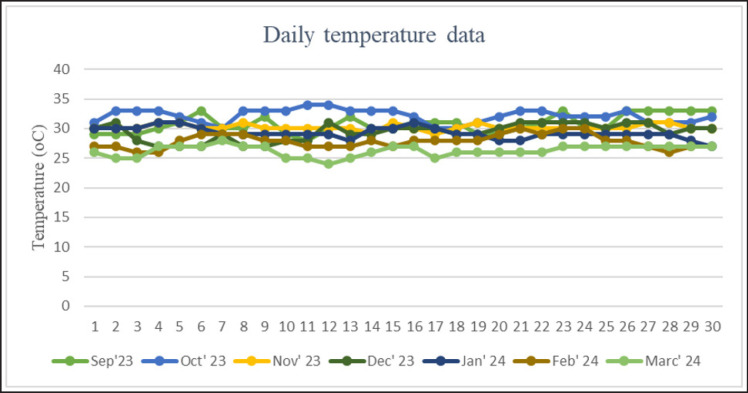
Daily temperature data for 5 months is divided into two seasons: summer (September–December) and rainy season (January–March).

**Figure 2.**
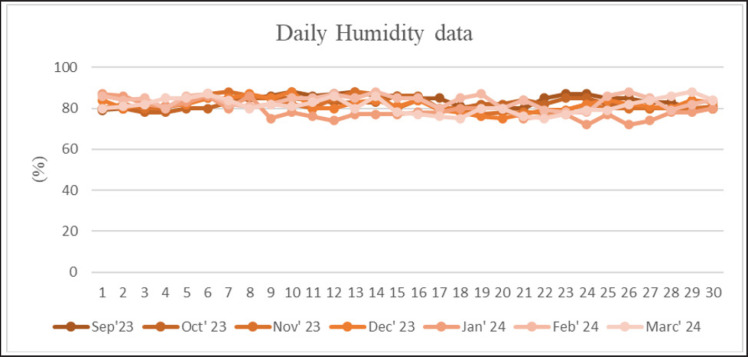
Daily humidity data for 5 months is divided into summer (September–December) and rainy seasons (January–March).

**Figure 3.**
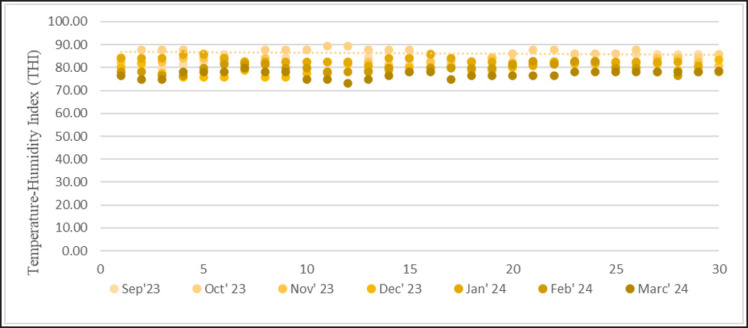
Daily THI data for 5 months is divided into two seasons: summer (September–December) and rainy season (January–March). The highest average THI on the axis line is in October.

Temperature, humidity, and THI for 7 months in Merauke from September 2023 to March 2024 are presented in Figures 1–3. The results showed that the average temperature in September was 30.73°C ± 1.53°C and humidity was 71.87°C ± 2.01°C. The increase in temperature in October reached 31.57°C ± 1.71°C, with humidity reaching 73.50°C ± 2.11°C. In November, the average temperature was 31.17°C ± 0.53°C, and humidity was 71.43°C ± 1.05°C. The average temperature in December reached 29.37°C ± 1.40°C, and humidity reached 77.67°C ± 1.26°C. In January–March 2024, there was a decrease in the average temperature of 29.23°C ± 0.60°C, 27.90°C ± 1.45°C, and 26.30°C ± 1.20°C. Humidity levels reached 78.77°C ± 2.24°C, 82.48°C ± 3.35°C, and 82.27°C ± 3.05°C. The THI value in summer is within the average danger level of 82.67°C ± 1.25°C. In the rainy season, the THI value ranges from 77.12°C ± 1.19°C to 81.47°C ± 1.33°C, which is the warning level of danger.

### Semen quality post-thawing

The study results are shown in Table 1, which shows that the quality of post-thawing conventional frozen semen is generally higher than that of sexing frozen semen. The difference in the average percentage of conventional frozen semen and sexing semen is that sperm motility is at 5.4%, sperm viability at 3.85%, sperm abnormality at 0.8%, membrane integrity at 3.45%, and acrosome integrity at 2.75%. The results of post-thawing semen quality testing between conventional semen and sexing semen were significantly different (*p < *0.05) for all parameters, including sperm motility, sperm viability, sperm abnormalities, sperm membrane integrity, and sperm acrosome.

### Fertilization rates using sexed and conventional semen in different seasons

Based on the results (Table 2), it showed that there is a difference between the use of sexed and conventional semen in different seasons. The CR value in the rainy season is significantly different (*p < *0.05) from the summer season, while the S/C value in the rainy season and summer is not significantly different (*p *> 0.05). Generally, the fertility rate in the rainy season was higher than in the summer. S/C and CR values using sexed semen and conventional semen in different seasons in this study were within the normal range.

**Table 1.** Quality of sexed and conventional semen post-thawing (*n* = 30, ± SE).

Characteristics	Fresh semen BC	Post thawing	
		Sexing	Conventional
Sperm motility Sperm viability Sperm abnormalities Sperm membrane integrity Sperm acrosome integrity	89.75 ± 0.63% 97.25 ± 0.03% 7.30 ± 1.40% 73.15 ± 0.15% 94.40 ± 0.76%	60.05 ± 0.8%^a^ 67.20 ± 0.7%^a^ 6.2 ± 1.2%^a^ 62.05 ± 0.6%^a^ 88.90 ± 0.8%^a^	65.45 ± 0.3%^b^ 71.05 ± 0.7%^b^ 5.4 ± 0.5%^b^ 65.5 ± 0.9%^b^ 91.65 ± 0.7%^b^

3BC: Brahman cross bull semen was collected using an artificial vagina, 15 semen conventional, 15 semen sexing, diluted using Andromed^®^, and frozen in 0.25 ml strow. Different letters (a,b) on the same row (*p < *0.05)

**Table 2.** Fertilization rates using conventional and sexed semen in different seasons ( ± SE).

Semen	Fertility rate	
	Dry season (32°C–34°C)	Rainy season (26.26°C–30.37°C)
Conventional frozen (CR)	75.00 ± 0.26^aC^	85.00 ± 1.03^bC^
Sexing frozen (CR)	65.00 ± 0.41^aC^	80.00 ± 0.20^bD^
Conventional frozen (S/C)	1.55 ± 0.49^a^	1.45 ± 0.47^a^
Sexing frozen (S/C)	1.62 ± 0.47^a^	1.55 ± 0.45^a^
Differences in fertilization rates	10%	5%

Different letters (a,b) in the same row (*p < *0.05), different letters (C, D) in the same column (*p < *0.05), (*n* = 80).

### Pregnancy in cows

**Figure 4.**
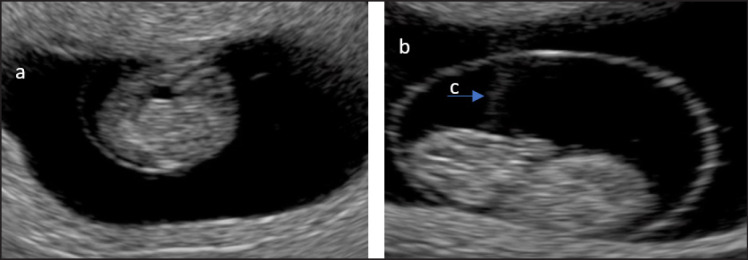
(a) Pregnancy 35 days, (b) pregnancy 55 days, and (c) placenta.

Pregnancy was identified at 35 and 55 days of age (Fig. 4). At 35 days of pregnancy, the average fetus diameter was 1.55 cm. At 35 days pregnant, a heartbeat was detected, and at 55 days, the fetus diameter averaged 4.85 cm. The embryo’s development will increase after more than 60–65 days. Detection of pregnancy and the use of ultrasound to provide an overview of the condition of the embryo in early pregnancy is essential. In general, reproductive disorders, especially miscarriages in livestock, occur early in pregnancy, so early detection will provide critical information for treatment and prevention.

## Discussion

Based on our hypothesis, environmental temperature outside and inside the shed and high humidity will generally affect the condition of livestock, especially their reproductive system. Typically, the critical temperature threshold in dairy cattle is 25°C, while in beef cattle, it is 30°C [9]. The higher the temperature and humidity, the higher the stress level. Stress in cattle impacts the increasing secretion of gonadotropin hormones [10]. In addition, several reproductive processes can be disturbed, such as embryo growth, gonadotropin secretion, ovarian follicle growth, and corpus luteum development [11]. Research conducted by Polsky and Keyserlingk et al. [12] shows that heat stress can be caused by increased environmental temperature and humidity, negatively affecting fertilization rates and milk production after AI. This condition certainly impacts the loss of economic value for breeders. The high THI level in this study at the warning-danger level generally impacted the CR. In addition, sexed semen decreased the fertilization rate compared to conventional semen. Nishisozu et al. [13] found that temperature affects the CR of heifers. Low CR can be caused by several factors related to the stress level of the estrous cycle and the suspected delay in ovulation. Cows experiencing heat stress tend to have small follicles that cause late ovulation [14]. THI values >75 in Merauke, South Papua conditions can trigger delayed estrus in female cattle and reduce CR. In addition, THI values >74–78 can cause increased stress in bulls and affect sperm quality [15].

Quality post-thawing motility (PTM) of sexed and conventional semen has differences. Still, both can be used for AI, based on the Indonesian National Standard in 2021 [16], frozen semen that posts thawing semen motility >40%. Another study stated that the PTM value of Ongole cattle sperm grade averaged 49%–62% [1]. The difference in PTM between sexed semen and conventional semen can occur because separating sperm to determine sex (X and Y) has an impact on sperm motility. This finding is reinforced by Steele et al. [17]: the sperm sexing process can reduce progressive sperm motility. Sexing techniques reduce sperm motility, viability, and fertilization capacity, with post-thawing quality of sperm sexing motility between 41% and 56% [18]. Semen sexing based on sex has the ability in the fertilization process, and viability is lower than conventional after the AI process [19].

The CR and S/C can indicate the fertilization rate. These conditions provide an overview of the success of the AI process and are a benchmark for AI officers. The success of S/C is related to the condition of estrus in cows. Using PGF2α plus vitamin E showed more evident signs of estrus and contributed to the AI process. The research results by Julanov et al. [20] showed that using PGF2α + Vitamin E + sodium selenite in synchronization showed estrus with a pregnancy rate reaching 100%. The addition of vitamin E in the synchronization process has a positive impact by improving reproductive function. Our results showed a CR rate of 85%, which was thought to be influenced by the THI level. The results of other studies state that season has little effect on the success of pregnancy in cows [21]. This condition may occur due to seasonal differences in South Papua-Indonesia with other regions. The use of sexed semen in different seasons does not affect S/C. Research by Yekti et al. [22] stated that the CR value of sexed semen was lower than that of conventional semen, with a difference of 10.23%. According to Oikawa et al. [23], the success rate of sexing semen is between 47%. In addition, the summer season has a higher S/C rate than the rainy season [24]. This condition is due to the stress level, which is related to the condition of the ewe’s follicles and estrus period [12]. Conventional (non-sexing) semen does not damage the S/C value because the freezing process does not go through a separation process like sexing semen. This study’s S/C and CR values were generally in average condition. According to Mutmaina et al. [25], the standard S/C value is between 1.6-2, and CR >60%.

Pregnancy detection in cattle can be done at 35 and 55 days of age. Detection of pregnancy can be done in various ways, such as rectal palpation or using ultrasound equipment. Pregnancy detection using ultrasound can be done 30 days after AI [26], and rectal palpation to determine how pregnancy can be done 40 days after AI [27]. According to Stratman et al. [28], the size of the embryo length of Holstein cows aged 33–45 averaged 1.63 cm. It is essential to detect pregnancy early so that the size of the embryo can be known and efforts to provide nutritional intake can be made when there is a delay in embryo development. The length of the embryo on days 35–40 depends on the type of cow and the nutritional intake given to the mother. Daneshi et al. [29] believe maternal nutrition significantly affects the growth and development of the fetus in the womb until after birth.

## Conclusion

The use of sexed semen and conventional semen can be done in the rainy season because the yield rate is more than 80%. Using sexed semen with high THI levels in the summer impacted CR (%) values but did not impact S/C values. Optimization of summer sexing semen should be done with a double dose to increase post-AI fertilization.

## List of abbreviations

AI, Artificial insemination; BC, Brahman cross; BPS, Statistical data center; PTM, Post thawing motility; SNI, Indonesian National Standard.
